# The emerging double-edged sword role of Sirtuins in the gastric inflammation-carcinoma sequence revealed by bulk and single-cell transcriptomes

**DOI:** 10.3389/fonc.2022.1004726

**Published:** 2022-10-17

**Authors:** Mengyang Wang, Chenxiao Bi, Hong Li, Lizhen Lu, Tao Gao, Panpan Huang, Chengxia Liu, Bin Wang

**Affiliations:** ^1^ Department of Immunology, Binzhou Medical University, Yantai, China; ^2^ Department of Gastroenterology, Binzhou Medical University Hospital, Binzhou, China; ^3^ Department of Pathology, Binzhou Medical University Hospital, Binzhou, China

**Keywords:** inflammation-carcinoma sequence, gastric cancer, histone modification, Sirtuins, histone deacetylase

## Abstract

Histone modification and the inflammation-carcinoma sequence (ICS) have been acknowledgedly implicated in gastric carcinogenesis. However, the extremum expression of some histone modification genes (HMGs) in intestinal metaplasia (IM) rather than GC obscures the roles of HMGs in ICS. In this study, we assumed an explanation that the roles of HMGs in ICS were stage specific. Bulk RNA-seq on endoscopy biopsy samples from a total of 50 patients was accompanied by reanalysis of a set of published single-cell transcriptomes, which cross-sectionally profiled the transcriptomic features of chronic superficial gastritis (SG), atrophy gastritis (AG), IM, and early gastric cancer (GC). Differential analysis observed significantly peaked expression of *SIRT6* and *SIRT7* at IM. Weighted correlation network analysis on bulk transcriptome recognized significant correlations between *SIRT1/6* and IM. The single-cell atlas identified one subgroup of B cells expressing high level of *TFF1* (*TFF1* ^hi^ naive B cell) that theoretically played important roles in defending microbial infection, while *SIRT6* displayed a positive correlation with *TFF1* ^low^ naive B cells. Moreover, gene set enrichment analysis at different lesions (SG-AG, AG-IM, and IM-GC) highlighted that gene sets contributing to IM, e.g., Brush Border, were largely enriched from co-expressing genes of Sirtuins (SIRTs) in AG-IM. Surveys of the genes negatively correlated with *SIRT6* in public databases considered *SIRT6* as tumor suppressors, which was confirmed by the cell proliferation and migration assays after transient transfection of *SIRT6* overexpression vector into AGS cells. All the above observations were then confirmed by serial section-based immunohistochemistry against Ki-67, MUC2, MUC5AC, p53, and SIRT6 on the endoscopic submucosal dissection tissue. By contrast, the expression of the other HMGs varied even opposite within same family. Taken together, this study preliminarily demonstrated the two-edged sword role of SIRTs in ICS and, by extension, showed that the roles of HMGs in ICS were probably stage specific. Our study may provide new insights into and attract attention on gastric prevention and therapy targeting HMGs.

## 1 Introduction

Gastric cancer (GC) ranks fifth in global cancer incidence and fourth in mortality in 2020 with an incidence rate of 5.6% and a mortality rate of 7.7% (1). Approximately 90% of gastric cancers are adenocarcinomas, which are mainly subdivided into two histologic subtypes, intestinal type, and diffuse type (2). Chronic inflammation largely orchestrates the tumor microenvironment and hence greatly contributes to GC carcinogenesis, particularly the intestinal-type GC (3–5). According to pathology and epidemiology evidence, intestinal-type GC rather than diffuse-type GC is usually triggered by chronic superficial gastritis (SG), atrophy gastritis (AG), intestinal metaplasia (IM), and dysplasia in that order, whose progress may stretch over decades (6, 7), indicating the crucial roles of the inflammation-carcinoma sequence in intestinal-type GC carcinogenesis.

Histone modifications, including methylation, acetylation, and phosphorylation, can alter the accessibility of transcription factors and RNA polymerase II to the DNA transcription sites and hence affect gene transcription (8). Current studies have illustrated that histone modification is an indispensable participant in chronic inflammation and cancer (9, 10). In GC, the roles of histone modification in development, malignancy, and prognosis have been frequently studied (11, 12), yet the roles might vary in the inflammation-carcinoma sequence. For example, while the increased histone deacetylases (HDACs) have been associated with increased invasion, distant metastatic potential, nodal metastases, and decreased overall survival (13), mRNA levels of HDACs from SG to GC peaked at IM (14). The H3K9 methyltransferases were recognized to be associated with the poor prognosis of gastric cancer (15), while decreased H3K9 di/trimethylation was detected in increased gastric and colonic inflammation (16). One proposed explanation is that the roles of histone modification in the inflammation-carcinoma sequence are staged, which at first requires expression profiles of histone modification genes (HMGs) as well as the co-expressed genes in the inflammation-carcinoma sequence. Additionally, the risk factors in GC carcinogenesis, e.g., *Helicobacter pylori* infection, family history, and diet, have been well studied (17). Several studies have uncovered the tight associations between *H. pylori* infection and histone modifications. For example, *H. pylori* could inhibit autophagic flux, promote its intracellular survival and colonization by downregulating deacetylase *SIRT1* (Wang, 18) and decrease the expression of tumor suppressor protein p27 through inhibition of histone acetylation within the p27 promoter (19). Yet, little is known about the associations between histone modifications and other risk factors in the inflammation-carcinoma sequence. In the context that only 3% of *H. pylori*-infected people developed GC (20), which emphasized the importance of other risk factors, the expression profiles of HMGs and the profiles of previous and life histories in the inflammation-carcinoma sequence were required again. However, little information was valid. Since it is almost impossible to clinically profile the inflammation-carcinoma sequence unless a large-scale longitudinal follow-up study spanning across decades is performed, a cross-sectional study involving SG, AG, IM, and early GC might be helpful.

In this study, we aimed to (1) cross-sectionally profile the transcriptomic features of SG, AG, IM, and early GC, accompanied by comprehensive previous and life history and pathological features, and (2) to explore the potential roles of HMGs in different lesions of the inflammation-carcinoma sequence. Bulk RNA-seq on the endoscopy biopsy samples from SG, AG, IM, and early GC patients was carried out to conduct the bulk RNA transcriptome, in addition to re-analysis on a set of published single-cell transcriptome. Downstream analysis including differential analysis, WGCNA, and GSEA was executed to explore the roles of HMGs in different lesions. Immunohistochemistry, Western blotting, public data survey, and cell proliferation and migration assays after transient transfection of SIRT6 overexpression vector into AGS cells provided additional evidence. Taken together, we put forward and preliminarily confirmed that the roles of HMGs in SG, AG, IM, and early GC were staged, whose issues need further discussion and may provide new insights into gastric prevention and therapy targeting HMGs.

## 2 Materials and methods

### 2.1 Biopsy sample collection and RNA and protein synchronous extraction

The study was approved by the Clinical Research Ethics Committees of Binzhou Medical University Hospital (Ethical certification number: KYLL-2021-02). A total of 50 gastric biopsy tissues in gastric antrum were sampled from 9 SG, 9 AG, 14 IM, 18 early intestinal-type GC patients from Gastrointestinal Endoscopy, Center of BinZhou Medical University Hospital. Biopsy tissues were obtained during endoscopy and were subsequently frozen immediately at −80°C. It is worth noting that the diagnosis was determined by two independent pathologists based on HE dyes and endoscopy diagnosis. None of the patients received preoperative chemotherapy or radiotherapy. Patients did not take antibiotics within 2 months prior to the collection of biopsy samples. All subjects provided informed consent for obtaining study specimens and completed the questionnaires collecting information about previous and life histories as shown in **Supplementary Materials 1** and **Supplementary Table 1**.

### 2.2 Bulk RNA-seq transcriptome library construction, sequencing, and single-cell RNA-seq transcriptome data

Total RNA and protein of the gastric mucosa were synchronously extracted by the ALLPure DNA/RNA/Protein Kit (CWBIO, Cat.CW0591S) according to the manual. The total RNA with RQN > 7 (Qsep 100, Bioptic, Taiwan, China) was processed into rRNA depletion, RNA fragmentation, cDNA synthesis, ending-repair and dA-tailing, adapter ligation, and library amplification to construct a transcriptome library using the VAHTS^®^ Universal V8 RNA-seq Library Prep Kit for Illumina (Vazyme Biotech Co., Ltd) according to the manual. The fragment distribution of libraries was profiled in Qsep 100. Subsequently, the libraries were quantified using qPCR and sent to Novogene (Beijing, China) for sequencing on the NovaSeq 6000 platform. The single-cell RNA-seq (scRNA-seq) transcriptomes were downloaded from GEO at the accession number GSE134520.

### 2.3 Comparative transcriptome analysis

#### 2.3.1 Preparation of Bulk RNA-seq transcriptome and scRNA-seq transcriptome

The raw reads of bulk RNA-seq data were filtered using fastp software (version 0.23.2) with default parameters (21). Subjunc aligner and featureCounts in Subread software (22) were employed to align the clean reads against the GRCh38 genome dataset and to construct the gene-sample count table, respectively. R software and the package DESeq2 (23) were employed in the differential analysis. Variance stabilizing transformation (VST) was applied to normalize the read counts for downstream analysis. The package Seurat (24) was employed to read the feature-count matrix and downstream analysis. Cells were flagged as poor-quality ones if they met one of the following thresholds: (1) number of features (nFeature_RNA) < 400 or nFeature_RNA > 7,000; (2) the number of reads (nCount_RNA) < 500 or nCount_RNA > 80,000; and (3) total percentages of mitochondrial genes > 20% or ribosomal genes > 40%. In addition, the singlet cells were identified with the DoubletFinder package (25). The dimension reduction, cell clustering, and cluster annotation were reproduced according to the original literature (26). The HMGs were surveyed in PubMed with the keywords “histone methylation” and “histone acetylation”, respectively. The HMGs were manually reviewed and summarized in **Supplementary Table 2**.

#### 2.3.2 Co-expressing genes of HMGs and their biological functions

The WGCNA package was employed to construct weighted gene co-expression network analysis on bulk RNA-seq transcriptome (27), when scRNA-seq transcriptome and the package “scWGCNA” with pseudo-cell method (50 cells in one pseudo-cell) were employed to perform single-cell WGCNA at the cell-type level (28). To reduce the computational complexity, the top 3,000 variable genes were selected according to variance across samples. The genes that displayed strong weighted correlation (WR > 0.1) with HMGs were extracted. To generally explore the associations among HMG expressions, life histories, and pathological features in the inflammation-carcinoma sequence, WGCNA on all samples was carried out based on bulk RNA-seq and scRNA-seq transcriptomes, respectively. Moreover, canonical correlation analysis (CCA) in Vegan package was additionally performed on bulk RNA-seq transcriptome to investigate the associations between transcriptomic features and life histories as well as associations between transcriptomic features and pathological features.

Subsequently, the inflammation-carcinoma sequence was divided into three lesions including SG to AG (SG-AG), AG to IM (AG-IM), and IM to early GC (IM-GC). Based on bulk RNA-seq, WGCNA was carried out to identify the co-expressing genes of HMGs at different lesions. The biological functions of HMG co-expressing genes were subsequently explored by gene set enrichment analysis (GSEA) against the Molecular Signatures Database (MSigDB 7.5.1) by clusterProfiler packages (version 4.4.2) (29, 30), with the purpose of reflecting the impacts of HMGs on the inflammation-carcinoma sequence.

#### 2.3.3 Analysis based on public databases

UALCAN, a comprehensive and interactive web resource for analyzing cancer omics data (31), was visited to explore the relationship between specific gene and clinical patient prognosis. The IHC images from the Human Protein Atlas, an online tool containing immune-histochemistry profiles for about 20 cancer types in 10 different sections, were extracted to compare the protein expression level of specific gene between the normal tissue and the GC tissue. TIMER is a comprehensive resource for systematical analysis of immune infiltrates across diverse cancer types (32). In this study, the Gene module in Tumor Immune Estimation Resource was applied to evaluate the correlations between the HMGs and immune cell infiltration.

### 2.4 Western blotting and immunohistochemistry

Protein samples were prepared and separated by SDS-PAGE gels, transferred onto PVDF membrane, and blocked with 10% milk. Then, blots were hybridized with the anti-H3K9ac and anti-GAPDH listed in **Supplementary Table 3**. Tanon (Shang Hai, China) was employed for chemical exposure of PVDF membranes. To highlight the continuous variation in *SIRT6* expression in the inflammation-carcinoma sequence, the intestinal-type early GC samples presenting pathological characteristics of SG, AG, IM, and early GC in one tissue were picked for immunohistochemistry. Histological sections were dewaxed and hydrated for IHC analysis. The sections were incubated at 4°C overnight in the specific dilution (**Supplementary Table 3**) with primary antibodies, followed by incubation with secondary antibody for 30 min at 37°C. Then, sections were stained with diaminobenzidine (DAB) chromogenic reagent and hematoxylin. After sealing, the slides were observed under a microscope.

### 2.5 Transient transfection and cell proliferation and migration assay

*SIRT6* overexpression vector and relative controls (pLenti-GIII-CMV-CBH-GFP-2A-Puro Kan) were purchased from Applied Biological Materials Inc. (Jiangsu, China). The AGS cells (CL-0022, Procell), a type of gastric cancer cell line, were seeded on six-well plates 24 h prior to transfection, at a density of 5.0 × 10^5^ cells/well. Transfections were performed using DNAfectin™ Plus Transfection Reagent according to the manufacturer’s instructions. After transfection for 24 h, the expressed GFP was observed through fluorescence microscopy, and then harvested for assay of cell proliferation and migration activity using an xCelligence RTCA Dual Plate (RTCA-DP) instrument. For migration assays, CIM-plate 16 (ACEA Biosciences) was used according to the manufacturer’s recommendations. Electrical impedance changes were measured at a gold microelectrode plated on the bottom of a membrane separating the upper and lower chambers. The lower compartment was supplemented with 10% FBS-containing medium for migration assay. A total of 5 × 10^4^ cells suspended in serum-free medium were supplemented to the upper compartment of the plate. For proliferation assays, 25,000 cells/well were seeded in commercial E-plate 16 (ACEA Biosciences). The impedance was recorded per 15 min for 36 h.

## 3 Results

### 3.1 Expression profiles of HMGs in the inflammation-carcinoma sequence

**Figure 1:**
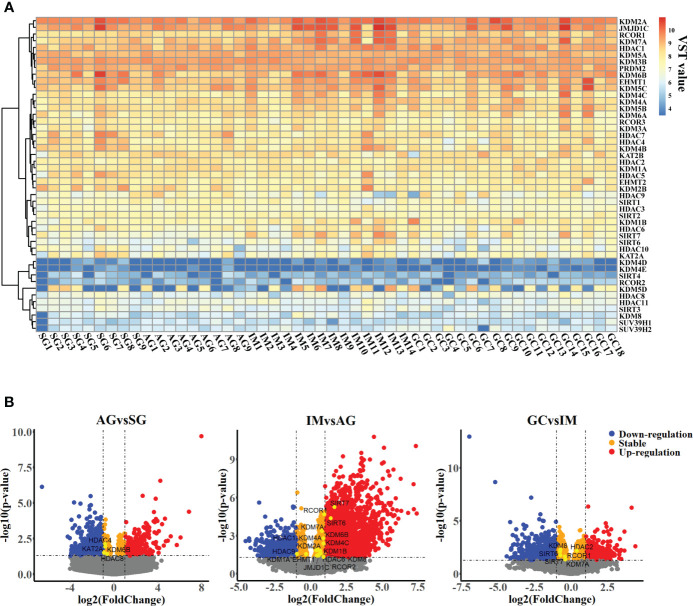
Transcriptomic profiles of gastric mucosa from patients suffering from chronic superficial gastritis, atrophy gastritis, intestinal metaplasia, and early gastric cancer. **(A)** Heatmap profiling variance stabilizing transformed expression (VST value) of histone modification genes across samples. **(B)** Volcano plot illustrating differential genes between adjacent groups.

**Figure 2:**
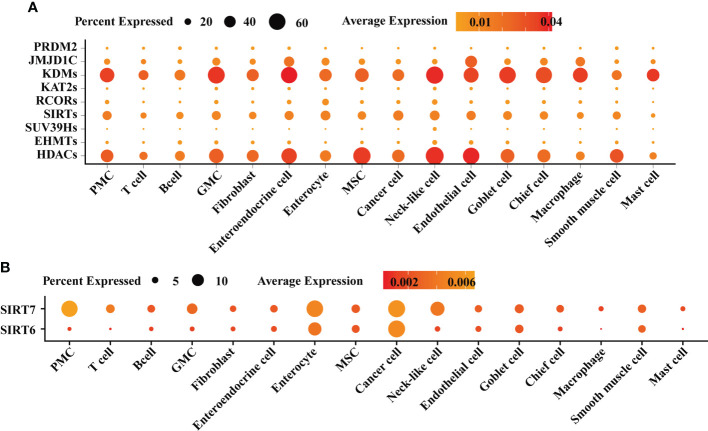
Single-cell atlas of gastric mucosae from patients suffering from chronic superficial gastritis, atrophy gastritis, intestinal metaplasia, and early gastric cancer. **(A)** Dot plot exhibiting the expression of histone modification genes across different cell types. **(B)** Dot plot exhibiting the expression of *SIRT6* and *SIRT7* across different cell types.

To profile the expression of HMGs in the inflammation-carcinoma sequence, bulk RNA-seq on biopsy tissues was carried out, in addition to reanalysis on a set of published single-cell transcriptome. In bulk RNA-seq datasets, 1,240,730,395 reads in 50,469 genes were detected in 50 biopsy specimens from 9 SG, 9 AG, 14 IM, and 18 early GC patients after filtering and aligning. As shown in **Figure S1**, PCA with a total variance of 66.31% in the first three axes (PC1 = 49.82%, PC2 = 11.33%, and PC3 = 5.16%) illustrated that the samples from SG and AG were grouped together while the samples from IM and GC were grouped together. As shown in **Figure 1A**, the family KDMs, HDACs, and SIRTs were the dominant HMGs expressed in the inflammation-carcinoma sequence. Differential analysis was performed on bulk RNA-seq datasets between adjacent lesions including AG vs. SG, IM vs. AG, and GC vs. IM (**Figure 1B**). The number of differential genes was highest with IM vs. AG, indicating that most noticeable changes in the inflammation-carcinoma sequence probably occurred from AG to IM. In addition, the expressions of *SIRT6* and *SIRT7* peaked at IM. In the scRNA-seq transcriptomes, 21,070 genes in 31,836 cells were detected. The T-SNE plot grouped enterocytes, chief cells, MSCs goblet cells, and cancer cells together (**Figure S2A**). From SG to GC, the percentages of MSCs and cancer cells were increased while enterocytes, goblet cells, and chief cells were decreased from IM to GC (**Figure S2B**). A dot plot of HMGs in all cell types illustrated that KDMs, HDACs, and SIRTs were the dominantly expressed HMGs (**Figure 2A**), showing consistency with the observation in our own bulk RNA-seq datasets. *SIRT6* and *SIRT7* were mainly expressed in cancer cells, enterocytes, MSCs, and goblet cells (**Figure 2B**), when *SIRT7* also expressed in PMCs and neck-like cells.

### 3.2 WGCNA revealed associations among HMG expressions, life histories, and pathological features in the inflammation-carcinoma sequence

**Figure 3:**
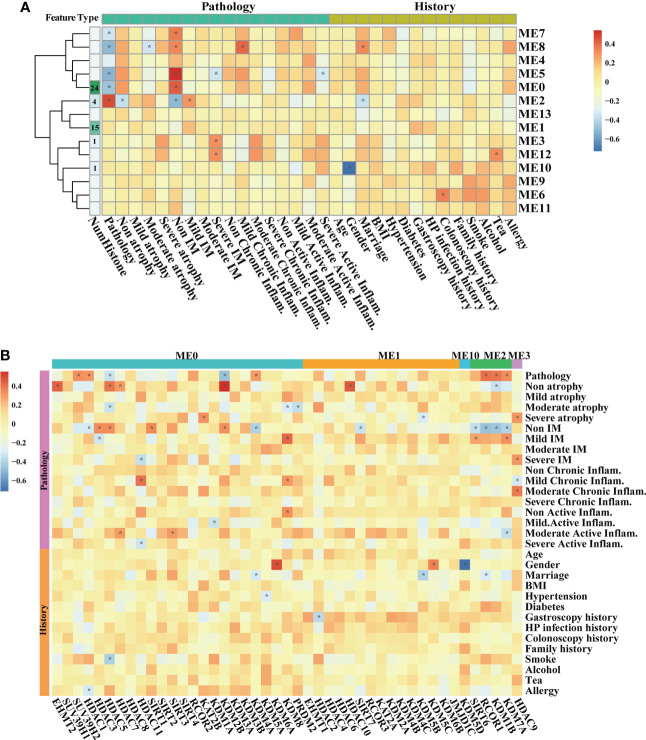
WGCNA on all samples based on bulk RNA-seq transcriptomes. Heatmaps illustrating modules–trait relationships **(A, B)** HMGs–trait relationships. The trait data were composed by diagnosis features in pathological sections, and previous history. The module affiliations of HMGs were labeled by the color bars on the top of the column. The symbol * means FDR adjusted p value for the labeled correlation was less than 0.05.

By involving all samples in one dataset, WGCNA was carried out to explore associations between HMG expressions and traits including life histories and pathological features in the inflammation-carcinoma sequence. The value of soft thresholding power was set to 20 to build a scale-free network (**Figure S3**). A total of 14 modules were detected. The module–trait relationships (**Figure 3A**) illustrated five modules (ME7, ME8, ME5, ME0, and ME2) displaying significant correlations with the “Pathology” feature of representing lesions. The atrophy features were negatively correlated with ME8 and ME2, when seven modules, particularly ME2, displayed significant correlations with IM. ME8 and ME5 were found to be positively correlated with mild chronic inflammation and negatively correlated with severe active inflammation, respectively. The HMGs were mainly distributed in ME0 (24 HMGs), ME1 (15 HMGs), ME2 (4 HMGs), ME3 (1 HMG), and ME10 (1 HMG) (**Figure 3B**), directing our attention to ME0 and ME2 and the HMGs embedded in them. In ME0, HDACs and KDMs were largely correlated with pathology, atrophy-related, and inflammation-related features, while *SIRT1* was correlated with “non. IM”, meaning no IM observed in the pathological section. In ME2, both *SIRT6* and *KDM7A* were negatively correlated with “non.IM” and were positively correlated with “mild.IM”, indicating their probable promoting roles in IM. CCA was additionally executed to examine the associations between life history and transcriptomes as well as between pathology features and transcriptomes (**Figure S4**). For the life history features, the first two axes explained the 39.86% of total variance, indicating that smoke, age, and *H*. *pylori* infection were the top three significant drivers associated with transcriptomic features. For the pathology features, moderate active inflammation, non.IM, and lesions were the top three significant drivers in the first two axes displaying 42.14% of total variance.

**Figure 4:**
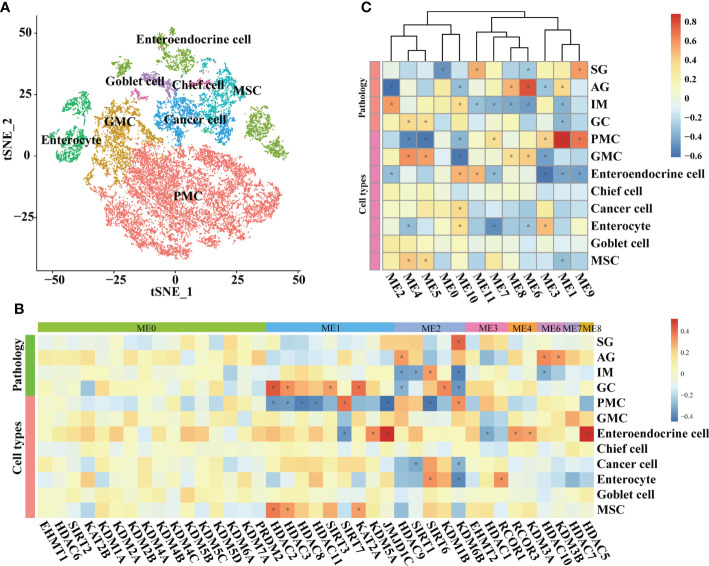
WGCNA on epithelial lineage cells based on scRNA-seq transcriptomes. **(A)** t-SNE plot visualizing epithelial lineage cells. Heatmaps illustrating modules–trait relationships **(B, C)** HMGs–trait relationships. The trait data were composed by lesions and cell types. The module affiliations of HMGs were labeled by the color bars on the top of the column. The symbol * means FDR adjusted p value for the labeled correlation was less than 0.05.

**Figure 5:**
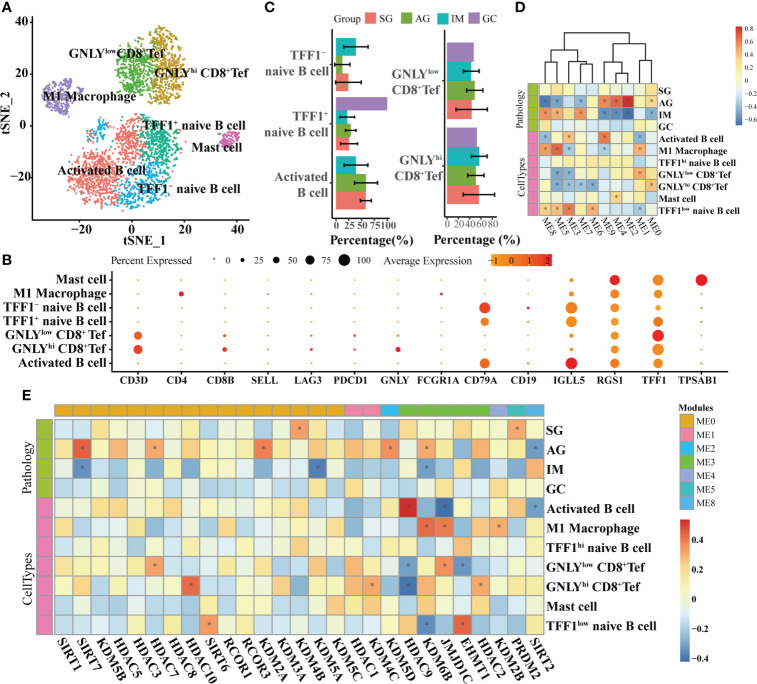
WGCNA on immune cells based on scRNA-seq transcriptomes. **(A)** t-SNE plot visualizing sub-grouped immune cells based on the expression of markers shown in **(B)**. **(C)** Bar plot illustrating the percentages of different immune cells in different lesions. Heatmaps illustrating modules–trait relationships **(D, E)** HMGs–trait relationships. The trait data were composed by lesions and cell types. The module affiliations of HMGs were labeled by the color bars on the top of the column. The symbol * means FDR adjusted p value for the labeled correlation was less than 0.05.

At the single-cell level, the associations between HMGs and pathology and cell types were explored in epithelial lineage cells and immune cells, respectively. In epithelial lineage cells, eight cell types were involved (**Figure 4A**). In the detected 12 modules, the HMGs were distributed in ME0, ME1, ME2, ME3, ME4, ME6, ME7, and ME8 modules (**Figure 4B**). Although ME0 was negatively correlated with SG (**Figure 4C**), none of the HMGs in ME0 displayed significant correlation with the traits. In contrast, ME1 and ME2 as well as the HMGs in the two modules exhibited significant correlations with the traits. The module ME1 was negatively correlated with “GC”, “MSC”, and “Enteroendocrine cell” and was positively correlated with “PMC”, while the HMGs in ME1, mainly functionally consisted of *HDAC2/3/8/11* and *SIRT3/7*, showed exact opposite trends to ME1. Remarkably, *SIRT6* and its harbor ME2 were both positively correlated with IM, indicating probable instrumental roles of *SIRT6* in IM again. In immune cells, the subtypes of different immune cells were firstly annotated according to the expression of markers (**Figures 5A, B**). As a result, the T cells were further annotated into GNLY ^low^- and GNLY ^hi^-CD8^+^ effector T cells (CD3D^+^, CD8B^+^, SELL^-^, and LAG3^-^), and B cells (CD79A^+^ and CD19^+^) were annotated into activated B cells (IGLL5^hi^ and RGS1^hi^), *TFF1* ^low^ naive B cells, and *TFF1* ^hi^ naive B cells. The percentage of activated B cells was decreased from AG to IM, displaying contrasting trends of the increased *TFF1* ^−^ naïve B cell (**Figure 5C**). A total of 10 modules were detected (**Figure 5D**), of which ME6/7/9 did not find distribution of HMGs (**Figure 5E**). ME0 accommodated the most HMGs, while ME3 displayed most correlations with immune cells, including negative correlations with M1 Macrophage and GNLY ^low^- and GNLY ^hi^-CD8^+^ effector T cells and positive correlations with activated B cells and TFF1^low^ naive B cells. Within SIRTs, only *SIRT6* displayed positive correlation with *TFF1* ^low^ naive B cells.

### 3.3 HMGs’ co-expressing genes at different lesions in bulk RNA-seq transcriptome

**Figure 6:**
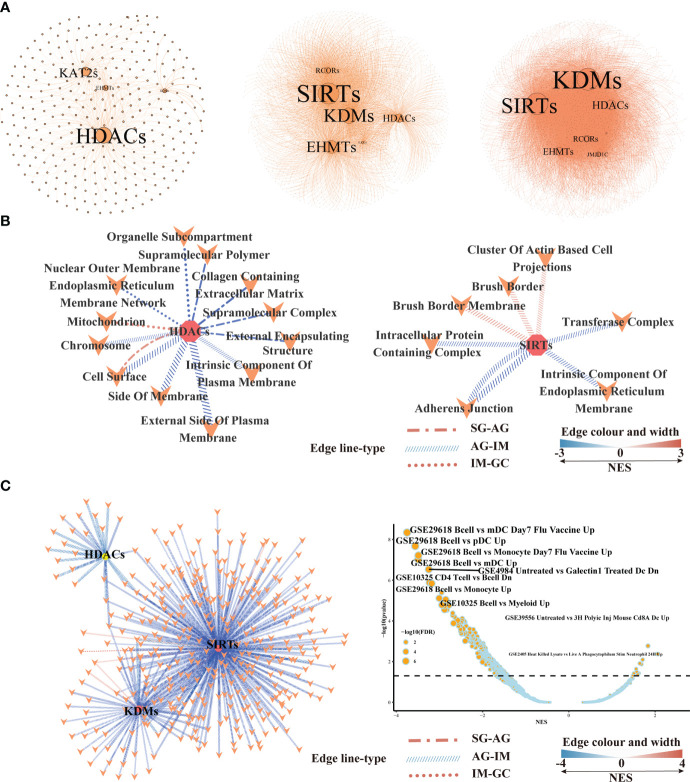
Gene set enrichment analysis based on the histone modification genes and their co-expressing genes in bulk RNA-seq transcriptome.**(A)** Networks illustrating co-expressing genes of histone modification genes at SG-AG (left), AG-IM (middle), and IM-GC (right). **(B)** Network showing the enriched gene sets based on the co-expressing genes of HDACs, SIRTs, and KDMs in different lesions. Only the significantly enriched gene sets against cellular component term in GO Ontology were shown here. Connection means significant enrichment. Edge line type represented the lesions, the color, and the width represented the scores of NES calculated by GSEA. **(C)** Network showing the enriched gene sets against immunologic signature database based on the co-expressing genes of HDACs, SIRTs and KDMs in different lesions. Connection means significant enrichment. Edge line type represented the lesions, the color, and the width represented the scores of NES calculated by GSEA.

At different lesions including SG-AG, AG-IM, and IM-GC, WGCNA respectively conducted 311, 1,859, and 2,576 nodes displaying high weighted correlations (*WR* > 0.1) with HMGs in the networks (**Figure 6A**). The degree of a node in a network, which was proportional to the node size, represented the number of co-expressing genes. HDACs and KATs displayed the top two high node degrees at the SG-AG lesion, while SIRTs and KDMs showed the top two high node degrees at AG-IM and IM-GC lesions. GSEA was employed to reveal the biological functions of genes in the networks. The network was employed to generally illustrate the number of significantly enriched down-/upregulated pathways at different lesions (**Figure S5**). The numbers of significantly enriched pathways SG-AG, AG-IM, and IM-GC were 1,146, 2,428, and 325, respectively, suggesting peaked importance of HMGs at AG-IM. In addition, most of the enriched gene sets in AG-IM were downregulated, whereas the enriched gene sets in SG-AG and IM-GC were largely upregulated. Combining everything we have found so far, further emphasis was laid on SIRTs, KDMs, and HDACs (**Figure 6B**). Using the co-expressing genes of the three families, a total of 21 gene sets were enriched against the Cellular Component (CC) term in GO Ontology. No significant gene set was enriched using KDMs co-expressing genes, nor was the gene set shared between HDACs and SIRTs, suggesting their distinct roles in the inflammation-carcinoma sequence. There were significant gene sets connected to HDACs in all lesions, whereas significant gene sets connected to SIRTs were only found in AG-IM, suggesting the stage-specific roles of SIRTs in AG-IM. Additionally, the three upregulated pathways including “Brush Border”, “Brush Border Membrane”, and “Cluster of Actin Based Cell Projections” in AG-IM lesion were connected to SIRTs, further suggesting potentially characteristic roles of SIRTs in AG-IM.

GSEA against “ImmuneSigDB” illustrated only one enriched gene set in IM-GC and AG-SG lesions based on HMGs’ co-expressing genes, whereas based on all genes, a total of 750 and 472 enriched pathways were detected in SG-AG and IM-GC, respectively (**Figure S6A**). Moreover, the HMGs were typed into TIMER web tool to explore the correlations between their expression levels and abundance of immune infiltrates (**Figure S6B**). With a cutoff value of |*partial.cor*| ≥ 0.1 and *p* < 0.05, few significant correlations were observed, indicating the possibly unimportant roles of SIRTs, HDACs, and KDMs in recruiting immune cells in IM-GC. However, many gene sets were downregulated in AG-IM based on HMGs’ co-expressing genes as shown in **Figure 6C**, of which a large part was connected to SIRTs. The most obvious changed gene sets were the downregulated B cell-related gene sets. Therefore, we preferred that the SIRTs acted AG-IM stage-specific roles in immune response in the inflammation-carcinoma sequence, showing consistency with previous observations in **Figure 5**.

### 3.4 Exploration on *SIRT6* by serial section-based immunohistochemistry, and transient transfection in AGS cells

**Figure 7:**
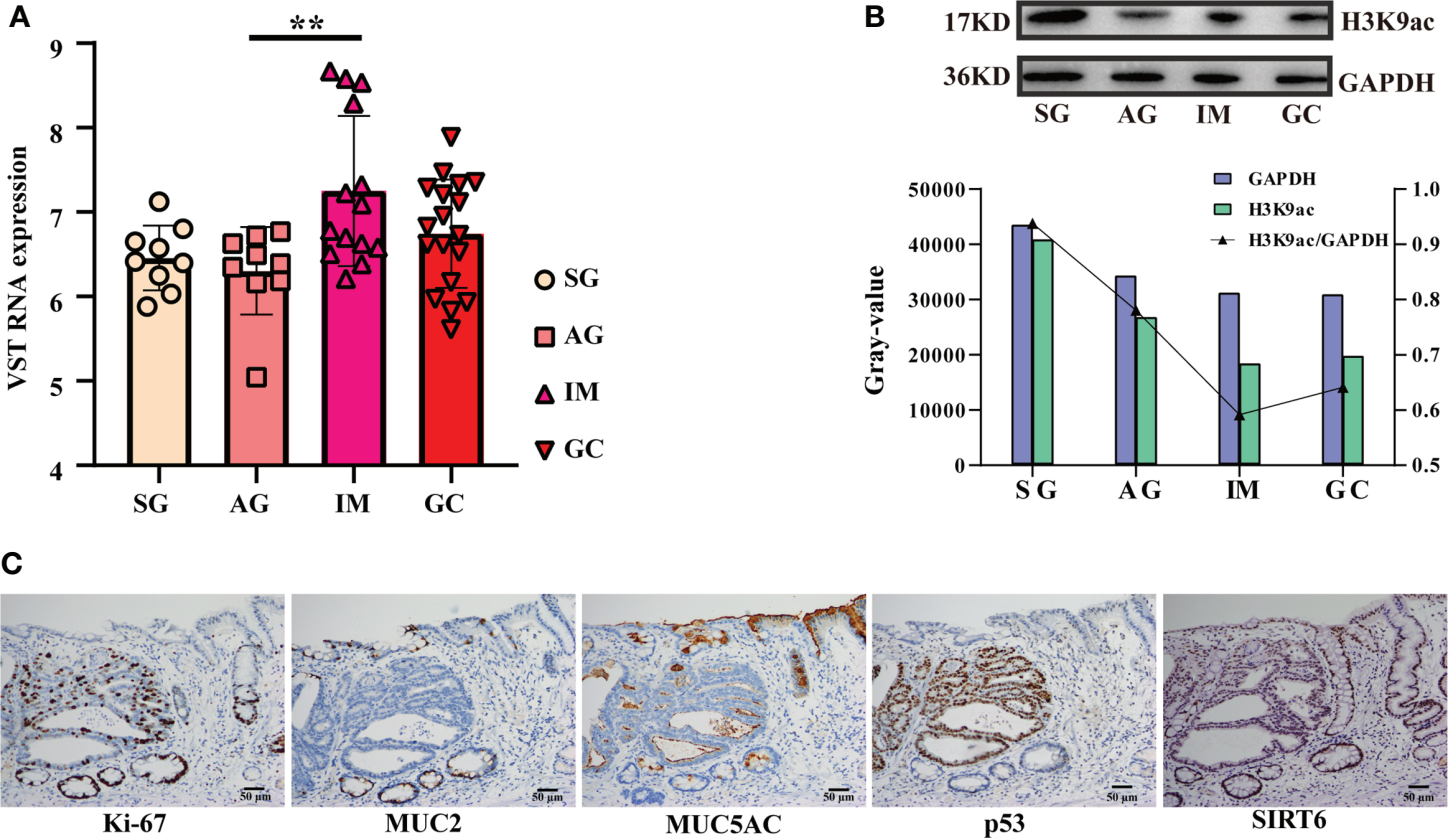
Detection and exploration on *SIRT6*. **(A)** Bar plot exhibiting the *SIRT6*’s expression from SG to the GC lesion. The symbol ** means FDR adjusted p value for the mean difference was less than 0.01 **(B)** Western blotting against H3K9ac(17KD) and GAPDH (36KD). The bar plot illustrating grayscale ratio of H3K9ac and GAPDH. **(C)** Immunohistochemistry against Ki-67, MUC2, MUC5AC, p53, and SIRT6 on an endoscopic submucosal dissection tissue.

In this study, we noted that *SIRT6* may play important roles in the inflammation-carcinoma sequence, particularly in IM. *SIRT6* showed peaked expression level at IM (**Figure 7A**). As *SIRT6* can mediate deacetylation of lysine 9 in histone H3, Western blotting against H3K9ac was first carried out to preliminarily examine if *SIRT6* worked. The results demonstrated minimum H3K9ac level along with the peaked *SIRT6* at IM (**Figure 7B**). Additionally, the GC tissue immunohistochemistry against Ki-67, MUC2, MUC5AC, p53, and SIRT6 on the serial sections of endoscopic submucosal dissection tissue provided hard evidence as well (**Figure 7C**). Ki-67 immunostaining was mainly distributed in IM, dysplasia, and cancer cells. MUC2 immunostaining was absent in normal gastric mucosa but consistently strong in IM glands. MUC5AC immunostaining was absent. p53 was predominantly stained in dysplasia and cancer cells. SIRT6 immunostaining exhibited decreased levels from IM, dysplasia, and normal gastric mucosa in that order. In addition, the UALCAN web tool was employed to investigate the potential involvements of *SIRT6* in GC carcinogenesis. Survival analysis indicated no significant association between *SIRT6* expression and survival rate (**Figure S7**). In addition, the genes meeting the thresholds, including (1) being core genes in cancer modules in Msigdb C4 gene sets and (2) displaying the top nine negative correlations with *SIRT6* across all samples, were involved in survival analysis as well (**Figure S8A, B**). In comparison with low expression levels, high expression levels of the nine genes showed a lower survival rate, consistent with the exploration of their immunostaining in control and GC tissues in the Human Protein Atlas (**Figure S9**), suggesting that SIRT6 might be a tumor suppressor.

**Figure 8:**
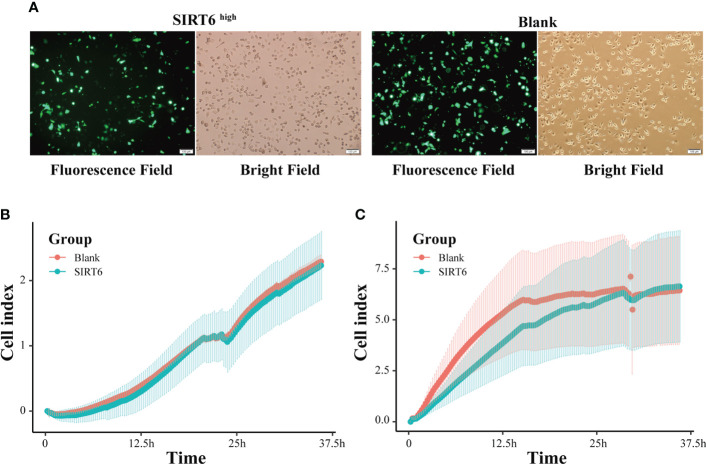
*SIRT6* overexpression inhibited GC cell proliferation. **(A)** AGS cells were examined by light microscopy and fluorescence microscopy 24 h after transfection with SIRT6 overexpression vector or blank vector. **(B)** Line chart showing AGS cells’ real-time migration in 36 h with cell index by RTCA assays. **(C)** Line chart showing AGS cells’ real-time proliferation in 36 h with cell index by RTCA assays.

To illuminate the influences of *SIRT6* on the GC cells, cell proliferation and migration assays were carried out using RTCA-DP after transient transfection of *SIRT6* overexpression vector into AGS cells for 24 h. As shown in **Figure 8A**, both *SIRT6* overexpression vector and blank vector have been efficiently transfected into AGS cells, and the vectors were highly expressed. For the proliferation, two groups, the SIRT6^high^ group and the Blank group, sheltered a similar number of AGS cells at the plateau phase (**Figure 8B**). However, the time taken to achieve the plateau phase was longer for the SIRT6^high^ group, which reached the plateau at 28 h, whereas the Blank group achieved the plateau at 15 h. For the migration, no significant difference was observed in 36 h (**Figure 8C**).

## 4 Discussion

### 4.1 SIRTs probably act as a two-edged sword in the inflammation-carcinoma sequence

Our results indicated probably staged roles of SIRTs, particularly *SIRT6*, in the intestinal-type gastric cancer inflammation-carcinoma sequence. SIRTs are the mammalian homologues of the yeast silent information regulator, which so far has identified seven members, from *SIRT1* to *SIRT7*. SIRTs share a highly conserved catalytic domain and NAD^+^ binding site, enabling their localization in different cellular compartments and their diverse physiological functions. SIRTs were usually recognized as cancer suppressors. *SIRT5* could enhance autophagy in GC cells through the mammalian AMPK-mTOR signaling pathway (33). *SIRT1* could inhibit proliferation and metastasis of gastric cancer cells *via* the STAT3/MMP-13 signaling pathway (34). In this study, WGCNA uncovered a negative correlation between *SIRT1* and cancer cells based on scRNA-seq transcriptome (**Figure 4B**), indicating its suppression on GC development. *SIRT6* was another uncovered member of SIRTs displaying associations with GC lesions, despite the fact that the role of *SIRT6* in gastric carcinogenesis was still unclear. *SIRT6* is primarily localized in the nucleus and has been identified to play essential roles in regulating metabolism, inflammation, and DNA repair (35). Current studies have identified its dual role of tumor suppression and promotion even in the same cancer. For example, *SIRT6* could promote EMT process in colon cancer (36), whereas *SIRT6* stabilization enabled inhibition by *USP10* (37). In this study, *SIRT6* exerted probable suppression in GC. First, its expression at mRNA and protein levels was both decreased from IM to GC (**Figures 1B**, **7A, C**), in agreement with the observation by Kugel et al. when they identified *SIRT6* as a pancreatic cancer suppressor (38). Additionally, *SIRT6* was supposed to exert inhibition on gene transcription *via* deacetylation of H3K9ac (35). H3K9ac has illuminated the importance of activating the transcription of multiple cancer-associated genes in different studies (39, 40). Second, we investigated the roles of nine representative genes that negatively correlated with *SIRT6* in GC. The higher expression in cancer compared with normal tissue, and the high expression associated with poor prognosis suggested the promotion of the nine genes in GC (**Figures S8A, B, S9**), allowing a large number of evidence to demonstrate their role in different cancers (18, 41–44). Third, to directly investigate the influences of *SIRT6* on GC cells, the transient transfection of *SIRT6* overexpression vector into AGS cells was carried out, followed by cell proliferation and migration assays. The results directly proved that overexpressed *SIRT6* inhibited the proliferation of AGS (**Figure 8**), showing agreement with a previous study (45).

On the other hand, the SIRTs appear to promote IM. The first evidence was that the expression of *SIRT6* was largely increased from AG to IM at both the mRNA and protein levels (**Figure 7**). In this study, immunohistochemistry on the intestinal-type early GC samples was applied to present all stages of the inflammation-carcinoma sequence in one tissue. According to previous studies, p53, MUC2, and MUC5AC represented dysplasia, intestinal gland, and gastric gland, respectively (46–49). We demonstrated that *SIRT6* was highly expressed in the IM glands where *SIRT6* immunostaining almost coincided with MUC2 immunostaining, whereas the *SIRT6* expression of adjacent AG and GC tissue is lower than IM. The only study by Liu et al. observed increased *SIRT6* in the gastric mucosa of Atp4a-/- mice that developed parietal cell atrophy and IM with elevated MUC2 expression, in agreement with this study (50). Second, WGCNA based on the bulk RNA-seq transcriptome of all samples revealed a negative correlation between *SIRT6/7* and non-IM but a positive correlation between *SIRT6* and mild-IM (**Figure 3B**), indicating the high correlation between *SIRT6* and IM. Furthermore, WGCNA at different lesions presented more strong evidence that the upregulated GO terms “Brush Border” and “Brush Border Membrane” were only connected to SIRTs at AG-IM lesion. The brush border is a highly specialized structure on the apical surface of enterocytes. It is well-adapted for efficient digestion and nutrient transport, and provides a protective barrier for the intestinal mucosa (51). The occurrence of brush border in gastric mucosa is hence an important feature of IM. To our knowledge, this study observed the potential associations between occurrence of brush border and SIRTs for the first time. Third, reanalysis on the published scRNA atlas from SG to early GC (34) presented additional evidence. *SIRT6* was mainly expressed by enterocytes and MSCs (**Figure 2**), which was confirmed by immunohistochemistry against *SIRT6* in this study (**Figure 7C**). WGCNA in epithelial lineage cells indicates a positive correlation of *SIRT6* with enterocyte in gastric mucosa (**Figure 5B**). In addition, inflammation and immune cells are key factors in IM. Chronic infection with *H. pylori* causes loss of parietal cells and acid production, which has been recognized as a key driver of IM in stomach (7). More emerging evidence demonstrated that the infection of microorganisms besides *H. pylori* played important roles in GC development as well (52, 53). The long-term and continuous microbial infection locally recruits lymphocytes and the secretion of cytokines, which subsequently influence epithelial cell signaling and the induction of metaplasia through various pathways (54). The key to this is long-term and sustainable infections, which may result from the loss of acid reduction, or lowered immune defense. Humoral immunity plays dominant roles in mucosal immunity against microbial infection (55). In this study, the percentage of activated B cell was decreased from AG to IM in the single-cell atlas (**Figure 5C**). Likewise, GSEA against ImmuneSigDB based on HMG co-expressing genes illustrated remarkably downregulated pathways connected to AG-IM, particularly the B cell-relevant pathways (**Figure 6**). Additionally, TFF1, a secreted protein, could prevent the development of a chronic inflammation by counteracting bacteria colonization or by impeding the IL6-STAT3 pro-inflammatory signaling axis (56, 57). In this study, we identified a subgroup of *TFF1* ^hi^ B cells for the first time, which theoretically played important roles in defending microbial infection, whereas *SIRT6* was detected to be positively correlated with *TFF1* ^low^ B cells (**Figure 5E**). In summary, the above observations revealed that SIRTs, particularly the *SIRT6*, might promote the development of IM from AG.

However, despite being controversial, it is more and more widely accepted that patients with IM were at a higher risk of gastric cancer (58). In this study, WGCNA and CCA both indicated that IM and chronic inflammation were key factors in distinguishing the samples (**Figure 3**). In the context that SIRTs might promote IM but suppress GC development, we preferred to consider SIRTs as a two-edged sword in the inflammation-carcinoma sequence, which requires more attention in the SIRTs-based clinical therapy of AG and IM and in the prevention of GC.

### 4.2 Associations between traits and HDACs and KDMs revealed their various roles in the inflammation-carcinoma sequence

The HDAC family was one of the dominant HMGs in the gastric inflammation-carcinoma sequence in this study. Currently, regarding SIRTs as a Class III HDAC, a total of 18 HDAC enzymes in four main classes based on their homology to yeast HDAC have been identified in mammalian cells (59). The relationships between HDACs and gastric cancer cells have been well studied, uncovering the HDACs-associated mechanisms in carcinogenesis including decreased gene transcription and autophagy, downregulation of p21, increased anti-apoptotic factors and cellular motility, and chemotherapy resistance, and promoted more de-differentiated cancer state (13). By contrast, the roles of HDACs in the inflammation-carcinoma sequence were less studied. We have indicated the promotion of SIRTs, Class III HDACs, on IM above, yet the other HDACs seemingly displayed various roles in the inflammation-carcinoma sequence in this study. First, based on all samples across the four lesions, WGCNA based on bulk RNA-seq revealed the exact opposite correlations with severe IM and moderate chronic inflammation between *HDAC9* and *HDAC11* (**Figure 3B**). This opposition was demonstrated by observations in the single-cell atlas as well (**Figure 4C**). Villagra et al. have revealed that *HDAC11* could inhibit IL-10 expression and induce inflammatory antigen-presenting cells (60), showing agreement with the positive correlation between *HDAC11* and mild chronic inflammation in some degree. Likewise, other couples of opposite HMGs within the same family included *HDAC1* and *HDAC3*, KDM2, and KDM5/1/7 in this study. However, research on such opposition between different HMGs as well as the roles of HMGs’ opposition in GC developments has been scarce, while inhibitors of HMGs especially HDACs have been used clinically for a wide variety of disorders.

### 4.3 Limitations and future works

However, our study has limitations. First, the antecedents of intestinal GC are not always SG, AG, IM, and dysplasia in that order; meanwhile, patients with SG, AG, and IM may never develop into the next stage. Although the intestinal-type early GC samples presenting different lesions of the inflammation-carcinoma sequence in one tissue were processed into immunohistochemistry to illuminate the continuous variation of *SIRT6* in this study, further research based on engineered mouse models can provide more direct evidence. In clinical practice, a large-scale follow-up longitudinal study recording the complete progression of the inflammation-carcinoma sequence will be a difficult but necessary undertaking. Second, SIRTs were supposed to play their roles epigenetically, whereas the underlying epigenetic mechanisms were not detailed in this study. Direct evidence on how SIRTs take part in gene expression regulation in the inflammation-carcinoma sequence still needs to be explored.

## 5 Conclusion

In this study, we reported a comprehensive dataset composed of whole transcriptome accompanied by previous and life history from SG, AG, IM, and early GC patients. Based on the dataset and a set of published single-cell transcriptome, the expression of HMGs in the intestinal-type gastric cancer inflammation-carcinoma sequence was profiled. The roles of HMGs in the inflammation-carcinoma sequence were probably staged, with demarcation at the transformation from AG to IM. The SIRTs encoding H3 deacetylases were considered as a two-edged sword in the inflammation-carcinoma sequence, while HDACs and KDMs played various roles in the inflammation-carcinoma sequence. Taken together, we put forward and preliminarily confirmed the staged roles of HMGs in the inflammation-carcinoma sequence and their associations with life histories, a topic that needs further discussion and may provide new insights into gastric prevention and therapy.

## Data availability statement

The data presented in the study are publicly available in the National Genomics Data Center (https://ngdc.cncb.ac.cn/) repository, accession number HRA002702.

## Ethics statement

The studies involving human participants were reviewed and approved by KYLL-2021-02. The patients/participants provided their written informed consent to participate in this study.

## Author contributions

BW, CL, and TH designed the experiment. MW, CB, and BW analyzed and orchestrated the data and wrote the manuscript. CL carried out the endoscopy. MW and CB collected the samples, sent out questionnaires, and performed experiments in laboratories. LL and HL diagnosed the samples and provided pathological information. Panpan Huang helped with the experiments in laboratories. TG helped with sample collection and questionnaire. All authors contributed to the article and approved the submitted version.

## Funding

Funding for this research was provided by the National Natural Science Foundation of China (31800110), GDAS’ Special Project of Science and Technology Development (2020GDASYL-20200104010), and Initial Project from Binzhou Medical University (50012304420).

## Acknowledgments

We thank Tao Hu from Department of Immunology, Binzhou Medical University for his contribution in experiment design. We thank Guoyan Wang from the Medical Laboratory in Yantai Affiliated Hospital of Binzhou Medical University for her suggestions in laboratory experiments not merely in this study.

## Conflict of interest

The authors declare that the research was conducted in the absence of any commercial or financial relationships that could be construed as a potential conflict of interest.

## Publisher’s note

All claims expressed in this article are solely those of the authors and do not necessarily represent those of their affiliated organizations, or those of the publisher, the editors and the reviewers. Any product that may be evaluated in this article, or claim that may be made by its manufacturer, is not guaranteed or endorsed by the publisher.

## Supplementary material

This section includes the bilingual version of the questionnaire, Table 1 listing detailed information orchestrated from the questionnaire, Table 2 listing the HMGs and references, Table 3 listing primary antibodies in Western blotting, **Figure S1** showed the principal component analysis on all samples. **Figure S2** showed the single cell atlas of gastric mucosae from patients suffering from chronic SG, AG, intestinal metaplasia, and early gastric cancer. **Figure S3** showed the soft-thresholding power analysis was used to obtain the scale-free fit index of network topology. **Figure S4** showed the associations between bulk RNA-seq transcriptome features and life history and pathology features. **Figure S5** illustrated the number of significantly enriched gene sets by Gene set enrichment analysis based on the histone modification genes and their co-expressing genes in bulk RNA-seq transcriptome. **Figure S6** showed the associations between HMGs and immune response. **Figure S7** showed survival analysis predicting the relationship between *SIRT6* expression and patient survival. **Figure S8** showed dot plots showing top 9 genes negatively correlated with *SIRT6* across all samples and survival analysis of top 9 genes negatively correlated with *SIRT6*. **Figure S9** showed the exploration of genes’ immunostaining in control and GC tissues in the Human Protein Atlas. The genes were those negatively correlated with *SIRT6* in **Figure 7**.
