# Supplementary material for: The emerging double-edged sword role of Sirtuins in the gastric inflammation-carcinoma sequence revealed by bulk and single-cell transcriptomes
Source: Front Oncol. 2022 Oct 17;12:1004726. doi: 10.3389/fonc.2022.1004726 (PMC9619065; doi:10.3389/fonc.2022.1004726)
Supplement: Supplementary file 1 [file Table_1.docx]

*Supplementary Material*

**Supplementary Materials 1**

**Chronic gastritis patient dietary questionnaire**

**1.Base situation**

1.Name：

2. Birth：

3.Telephone：

4. Home Address：

5. Number of families：

6.Sex：□Male □Female

7.Marriage：□Unmarried □Married □Divorce □Bereft of one's spouse

8.Blood type：□A □ B □O □AB □Unknown

9. Ethnic groups：□Han □Uygur □ Hui □Other

**2.Healthy condition**

1.Height: cm; Weight: kg；

2. Disease duration： Week Month Year

Diagnostic methods：□Gastroscope □Inquiry

3.Emotional state：□Fine □Badly □Anxious □Depressed

4. Blood pressure: ①Yes Blood: mmol/Hg

② No

5. Diabetes mellitus：①Yes Blood sugar: mmol/L

②No

7. Dietary supplement

8. History and type of disease：

□Peptic ulcer □Gastric cancer □Helicobacter pylori infection □hepatopathy/hepatocirrhosis □Dental ulcer □Esophagitis □Coronary disease □Thyroid nodule □Blood system disease

□Other Time： Year Month

9. Whether the following diseases are present in immediate family members (parents, brother and sister)

□Atrophic gastritis □Chronic non-atrophic gastritis □Helicobacter pylori infection □ Peptic ulcer □Gastric cancer □Dental ulcer) □Esophagitis □Coronary disease □Blood system disease

Other：（Disease： ；Relation ）

**3.Food habits**

1. Smoking recently □Yes Time：____

□No □Has quit smoking Time：____

2. Drinking alcohol recently □Yes Time：____；Kind：____； □No □Has quit the wine Time：____

3. Possess hobby of Drinking tea □Yes □No

4. How often drinking tea

①Never drink ②Drink little ③Eat every day ④Once a week ⑤More than 1 time a week ⑥1次Eat once a month⑦ More than one time a month

**4.Dietary survey**

**4.1.Cereal food, beans, starch food staple food**

| Food name | Whether to eat  （Yes/No） | The frequency of eating | | | | Amount  （g） |
| --- | --- | --- | --- | --- | --- | --- |
|  |  | Time / Day | Time / Week | Time /Month | Time /Year |  |
| 1、Rice |  |  |  |  |  |  |
| 2. Steamed bun |  |  |  |  |  |  |
| 3. Millet congee/ White rice porridge |  |  |  |  |  |  |
| 4、Steamed bun / Dumpling |  |  |  |  |  |  |
| 5. Noodles |  |  |  |  |  |  |
| 6. Sweet potato |  |  |  |  |  |  |
| 7. Bean curd |  |  |  |  |  |  |
| 8.Soya-bean milk |  |  |  |  |  |  |
| 9. Other soy products  （ ） |  |  |  |  |  |  |

**4.2. Fresh vegetables**

| Food name | Whether to eat  （Yes/No） | The frequency of eating | | | | Amount  （g） |
| --- | --- | --- | --- | --- | --- | --- |
|  |  | Time / Day | Time / Week | Time /Month | Time /Year |  |
| 1. Spinacia oleracea |  |  |  |  |  |  |
| 2. Cabbage |  |  |  |  |  |  |
| 3. Bean sprout |  |  |  |  |  |  |
| 5. Broccoli |  |  |  |  |  |  |
| 6. Benincasa hispida |  |  |  |  |  |  |
| 7. Tomato |  |  |  |  |  |  |
| 8.Daucus carota |  |  |  |  |  |  |
| 9. Celery |  |  |  |  |  |  |
| 10. Agaric |  |  |  |  |  |  |
| 11. Pumpkin |  |  |  |  |  |  |
| 12. Cauliflower |  |  |  |  |  |  |
| 13. French bean |  |  |  |  |  |  |
| 14. Lotus root |  |  |  |  |  |  |
| 15. Onion |  |  |  |  |  |  |
| 16. Potato |  |  |  |  |  |  |
| 17.Chinese cabbage |  |  |  |  |  |  |
| 18.Crowndaisy chrysanthemum |  |  |  |  |  |  |
| 20.Rape |  |  |  |  |  |  |
| 21.Chinese yam |  |  |  |  |  |  |
| 22.Eggplant |  |  |  |  |  |  |
| 23.Fragrant-flowered garlic |  |  |  |  |  |  |
| 24.Pleurotus eryngii |  |  |  |  |  |  |
| 25.Shiitake |  |  |  |  |  |  |
| 26.Kidney bean |  |  |  |  |  |  |

**4.3.Livestock**

| Food name | Whether to eat  （Yes/No） | The frequency of eating | | | | Amount  （g） |
| --- | --- | --- | --- | --- | --- | --- |
|  |  | The frequency of eating | Time / Week | Time /Month | Time /Year |  |
| 1. Pork |  |  |  |  |  |  |
| 2. Brawn、Fish、Pork |  |  |  |  |  |  |
| 3.Pork liver |  |  |  |  |  |  |
| 4.Pig large intestine |  |  |  |  |  |  |
| 5.Pig kidneys |  |  |  |  |  |  |
| 6. Pausage |  |  |  |  |  |  |
| 7.Mutton |  |  |  |  |  |  |
| 8.Beef |  |  |  |  |  |  |
| 9.Chicken |  |  |  |  |  |  |
| 10.Duck |  |  |  |  |  |  |
| 12.Duck's egg（Salty） |  |  |  |  |  |  |
| 13.Egg |  |  |  |  |  |  |
| 14.Pidan |  |  |  |  |  |  |
| 15.Other  （ ） |  |  |  |  |  |  |

**4.4.Fruit**

| Food name | Whether to eat  （Yes/No） | The frequency of eating | | | | Amount  (g) |
| --- | --- | --- | --- | --- | --- | --- |
|  |  | The frequency of eating | Time / Week | Time /Month | Time /Year |  |
| 1.Apple |  |  |  |  |  |  |
| 2.Pear |  |  |  |  |  |  |
| 3.Orange |  |  |  |  |  |  |
| 4. Peach |  |  |  |  |  |  |
| 5. Watermelon |  |  |  |  |  |  |
| 6. Hami melon |  |  |  |  |  |  |
| 7. Banana |  |  |  |  |  |  |
| 8. Grape |  |  |  |  |  |  |
| 9. Yangtao |  |  |  |  |  |  |
| 10. Other  （ ） |  |  |  |  |  |  |

**4.5.Pickled vegetables**

| Food name | Whether to eat  （Yes/ No） | The frequency of eating | | | | Amount  (g) |
| --- | --- | --- | --- | --- | --- | --- |
|  |  | The frequency of eating | Time / Week | Time /Month | Time /Year |  |
| 1. Hot pickled mustard tuber |  |  |  |  |  |  |
| 2. Radish strip |  |  |  |  |  |  |
| 3. Pickled cucumbers |  |  |  |  |  |  |
| 10. Other  （ ） |  |  |  |  |  |  |

**4.6. Milk and dairy products**

| Food name | Whether to eat  （Yes/No） | The frequency of eating | | | | Amount  (g) |
| --- | --- | --- | --- | --- | --- | --- |
|  |  | The frequency of eating | Time / Week | Time /Month | Time /Year |  |
| 1. Cream |  |  |  |  |  |  |
| 2. Fresh milk |  |  |  |  |  |  |
| 3. Yoghourt |  |  |  |  |  |  |
| 10. Other  （ ） |  |  |  |  |  |  |

**5. 24-hour meal review**

Number： Name： Date：

| Project | Dish | Food composition | Weigh（g） |
| --- | --- | --- | --- |
| Breakfast |  |  |  |
| Lunch |  |  |  |
| Dinner |  |  |  |
| Add meals or snacks |  |  |  |

1. **Eortc QLQ-STO 22 questionnaire**

| Whether have syndrome as following in last week | | Score | | | | | | |
| --- | --- | --- | --- | --- | --- | --- | --- | --- |
|  |  | Not | A little | Bit | Medium | Pronounced | Severe | Very Severe |
| 1 | Epigastric pain | 1 | 2 | 3 | 4 | 5 | 6 | 7 |
| 2 | Dysphagi | 1 | 2 | 3 | 4 | 5 | 6 | 7 |
| 3 | Low food or soft food is difficult | 1 | 2 | 3 | 4 | 5 | 6 | 7 |
| 4 | Drinking water is difficult | 1 | 2 | 3 | 4 | 5 | 6 | 7 |
| 5 | Eating is all too comfortable | 1 | 2 | 3 | 4 | 5 | 6 | 7 |
| 6 | Have a stomachache | 1 | 2 | 3 | 4 | 5 | 6 | 7 |
| 7 | Stomach is not comfortable | 1 | 2 | 3 | 4 | 5 | 6 | 7 |
| 8 | Abdominal distension | 1 | 2 | 3 | 4 | 5 | 6 | 7 |
| 9 | Sour regurgitation | 1 | 2 | 3 | 4 | 5 | 6 | 7 |
| 10 | Stomach burning feeling | 1 | 2 | 3 | 4 | 5 | 6 | 7 |
| 11 | Often hiccups | 1 | 2 | 3 | 4 | 5 | 6 | 7 |
| 12 | Just eat to feel very full | 1 | 2 | 3 | 4 | 5 | 6 | 7 |
| 13 | Apocleisis | 1 | 2 | 3 | 4 | 5 | 6 | 7 |
| 14 | Eating takes a very long time | 1 | 2 | 3 | 4 | 5 | 6 | 7 |
| 15 | Not comfortable eating in front of others | 1 | 2 | 3 | 4 | 5 | 6 | 7 |
| 16 | Dry | 1 | 2 | 3 | 4 | 5 | 6 | 7 |

Recorder： Date：

**Supplementary Table 2** The genes capable in Histone modification

| **Family** | **Gene** | **Modification** | **Ref.** |
| --- | --- | --- | --- |
| **EHMT** | **EHMT1(GLP)** | Methylation | ^(Kramer, Kochinke et al. 2011)^ |
|  | **EHMT2(G9a)** | Methylation | ^(Kramer, Kochinke et al. 2011, Li, Zheng et al. 2019)^ |
| **SUV39H** | **SUV39H1(KMT1A)** | Methylation | ^(Li, Zheng et al. 2019)^ |
|  | **SUV39H2(KMT1B)** | Methylation | ^(Piao, Nakakido et al. , Piao, Yuan et al. 2019)^ |
| **HDAC** | **HDAC1** | Deacetylation | ^(Witt, Deubzer et al. 2009)^ |
|  | **HDAC2** | Deacetylation | ^(Witt, Deubzer et al. 2009)^ |
|  | **HDAC3** | Deacetylation | ^(Witt, Deubzer et al. 2009)^ |
|  | **HDAC4** | Deacetylation | ^(Witt, Deubzer et al. 2009)^ |
|  | **HDAC5** | Deacetylation | ^(Witt, Deubzer et al. 2009)^ |
|  | **HDAC6** | Deacetylation | ^(Witt, Deubzer et al. 2009)^ |
|  | **HDAC7** | Deacetylation | ^(Witt, Deubzer et al. 2009)^ |
|  | **HDAC8** | Deacetylation | ^(Witt, Deubzer et al. 2009)^ |
|  | **HDAC9** | Deacetylation | ^(Witt, Deubzer et al. 2009)^ |
|  | **HDAC10** | Deacetylation | ^(Witt, Deubzer et al. 2009)^ |
|  | **HDAC11** | Deacetylation | ^(Witt, Deubzer et al. 2009)^ |
| **SIRT** | **SIRT1** | Deacetylation | ^(Carafa, Rotili et al. 2016, Shen, Li et al. 2017, Schizas, Mastoraki et al. 2020)^ |
|  | **SIRT2** | Deacetylation | ^(Carafa, Rotili et al. 2016, Shen, Li et al. 2017, Schizas, Mastoraki et al. 2020)^ |
|  | **SIRT3** | Deacetylation | ^(Carafa, Rotili et al. 2016, Shen, Li et al. 2017)^ |
|  | **SIRT4** | Deacetylation | ^(Carafa, Rotili et al. 2016, Schizas, Mastoraki et al. 2020)^ |
|  | **SIRT5** | Deacetylation | ^(Carafa, Rotili et al. 2016, Shen, Li et al. 2017)^ |
|  | **SIRT6** | Deacetylation | ^(Carafa, Rotili et al. 2016, Shen, Li et al. 2017, Kuang, Chen et al. 2018, Schizas, Mastoraki et al. 2020)^ |
|  | **SIRT7** | Deacetylation | ^(Carafa, Rotili et al. 2016, Shen, Li et al. 2017, Schizas, Mastoraki et al. 2020)^ |
| **RCOR** | **RCOR1** | Deacetylation Inhibitor | ^(Rivera, Lee et al. 2022)^ |
|  | **RCOR2** | Demethylation Inhibitor | ^(Yang, Wang et al. 2011, Wang, Wu et al. 2016)^ |
|  | **RCOR3** | Demethylation Inhibitor | ^(Upadhyay, Chowdhury et al. 2014)^ |
| **KAT2** | **KAT2A(GCN5)** | Acetylation | ^(Allis, Berger et al. 2007, Fournier, Orpinell et al. 2016)^ |
|  | **KAT2B(PCAF)** | Acetylation | ^(Allis, Berger et al. 2007, Fournier, Orpinell et al. 2016)^ |
| **KDM** | **KDM1A(LSD1)** | Demethylation | ^(Wang, Wu et al. 2016)^ ^(Allis, Berger et al. 2007)^ |
|  | **KDM1B** | Demethylation | ^(Ciccone, Su et al. 2009)^ |
|  | **KDM2A** | Demethylation | ^(Allis, Berger et al. 2007)^ |
|  | **KDM2B** | Demethylation | ^(Allis, Berger et al. 2007)^ |
|  | **KDM3A** | Demethylation | ^(Allis, Berger et al. 2007)^ |
|  | **KDM3B** | Demethylation | ^(Allis, Berger et al. 2007)^ |
|  | **KDM4A** | Demethylation | ^(Allis, Berger et al. 2007)^ |
|  | **KDM4B** | Demethylation | ^(Allis, Berger et al. 2007)^ |
|  | **KDM4C** | Demethylation | ^(Allis, Berger et al. 2007)^ |
|  | **KDM4D** | Demethylation | ^(Allis, Berger et al. 2007)^ |
|  | **KDM4E** | Demethylation | ^(Thalhammer, Mecinovic et al. 2011)^ |
|  | **KDM5A** | Demethylation | ^(Allis, Berger et al. 2007)^ |
|  | **KDM5B** | Demethylation | ^(Allis, Berger et al. 2007)^ |
|  | **KDM5C** | Demethylation | ^(Allis, Berger et al. 2007)^ |
|  | **KDM5D** | Demethylation | ^(Allis, Berger et al. 2007)^ |
|  | **KDM6A** | Demethylation | ^(Allis, Berger et al. 2007)^ |
|  | **KDM6B** | Demethylation | ^(Allis, Berger et al. 2007)^ |
|  | **KDM7A** | Demethylation | ^(Huang, Xiang et al. 2010)^ |
|  | **KDM8** | Demethylation | ^(Wilkins, Islam et al. 2018)^ |
|  | **JMJD1C** | Demethylation | ^(Katoh and Katoh 2007)^ |
|  | **PRDM2** | Methylation | ^(Pandzic, Rendo et al. 2017)^ |

**Supplementary Table 3**

Antibodies used in the Western blotting and immunohistochemistry

|  | **Antibody** | **Company** | **Host** | **Dilution ratio** |
| --- | --- | --- | --- | --- |
| **WB**  **primary**  **antibodies** | H3K9ac | abcam [Y28] | Rabbit | 1:500 |
|  | H3K9ac | ABclonal [A7255] | Rabbit | 1:1000 |
|  | GAPDH | Abmart [M2006S] | Mouse | 1:5000 |
| **WB**  **secondary**  **antibodies** | Anti-Rabbit | Abmart[M21001S] | Goat | 1:5000 |
|  | Anti-Mouse | Abmart[M21002S] | Goat | 1:5000 |
| **IHC**  **primary**  **antibodies** | p53 | ZSGB-BIO[ZM-0408] | Mouse | 1:200 |
|  | Ki-67 | ZSGB-BIO[ZM-0166] | Mouse | 1:200 |
|  | MUC2 | MXB[MAB-0075] | Mouse | Ready-to-use |
|  | MUC5AC | MXB[MAB-0079] | Mouse | Ready-to-use |
|  | SIRT6 | abcam [EPR26255-85] | Rabbit | 1:2000 |
| **IHC**  **secondary**  **antibodies** | Enzyme conjugated Goat  anti-Mouse rabbit IgG polymer | ZSGB-BIO[PV-6000] | Goat | Ready-to-use |

**Supplementary Figures**

**
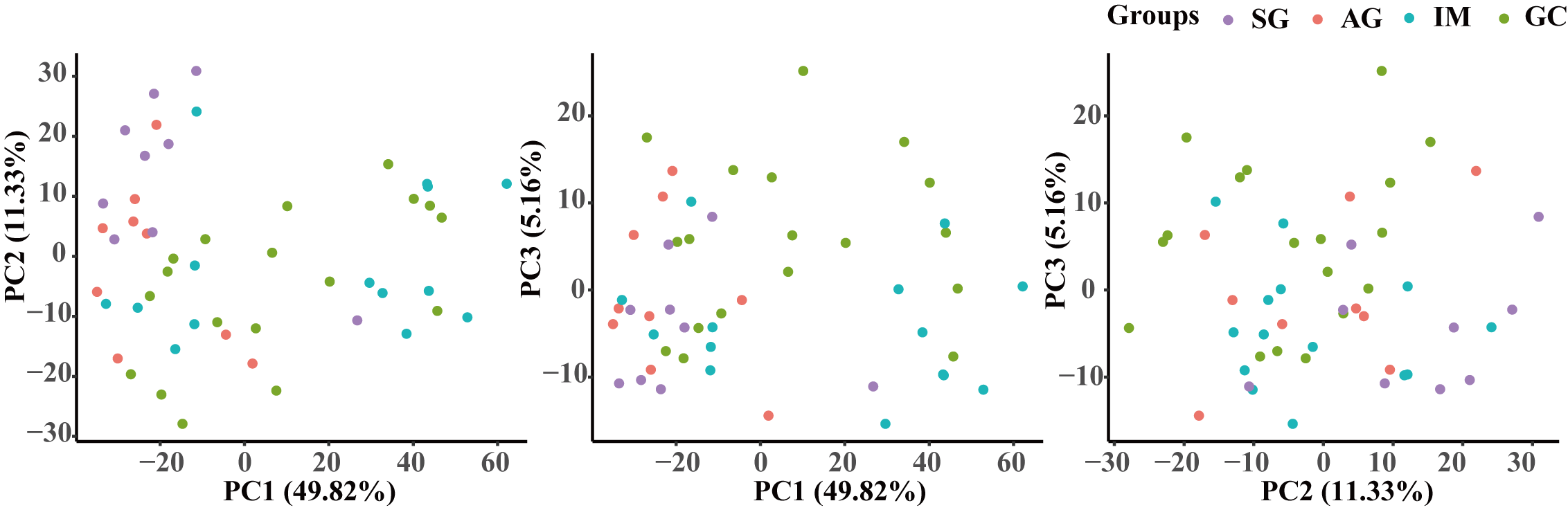
**

**Figure S1.** Principal component analysis on all samples.

**
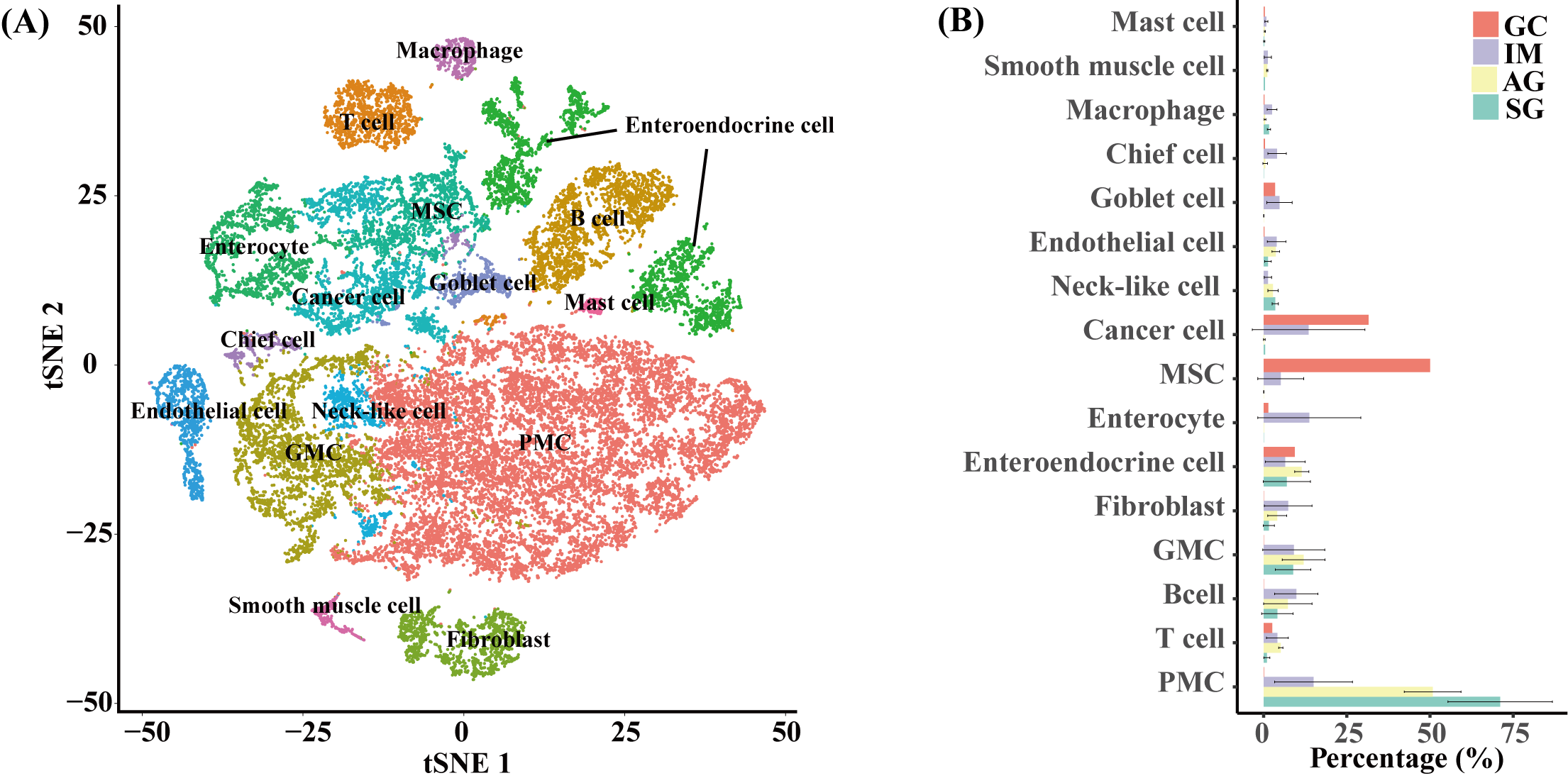
**

**Figure S2.** (A) t-SNE plot of 31,836 cells to visualize cell-types based on the expression of known markers. (B) Bar plot illustrating the percentages of different cell-types in different lesions.

**
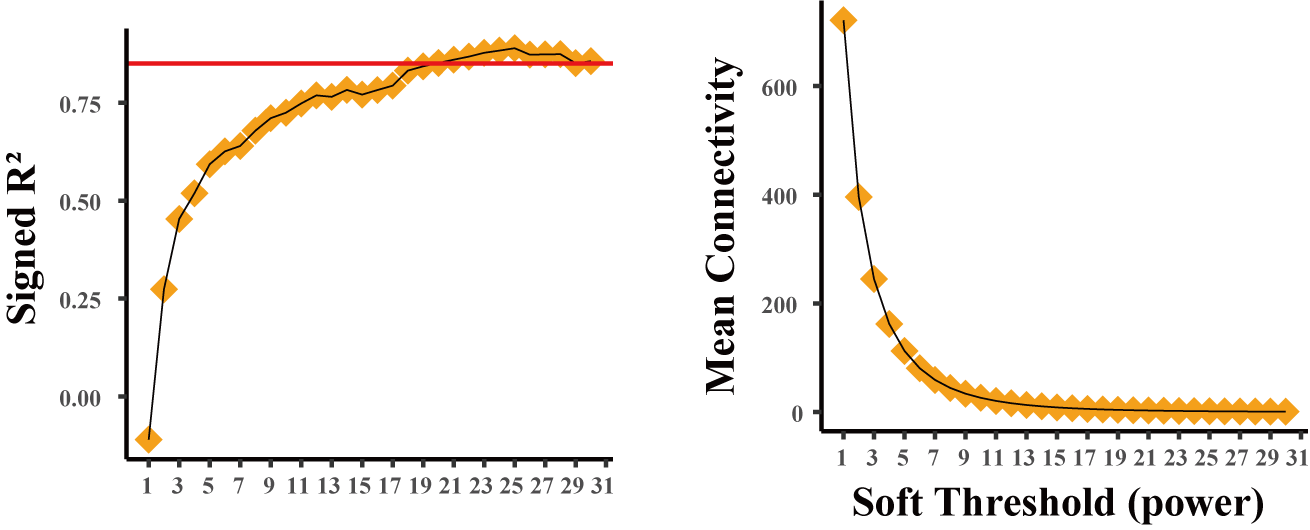
**

**Figure S3.** Soft-thresholding power analysis was used to obtain the scale-free fit index of network topology.


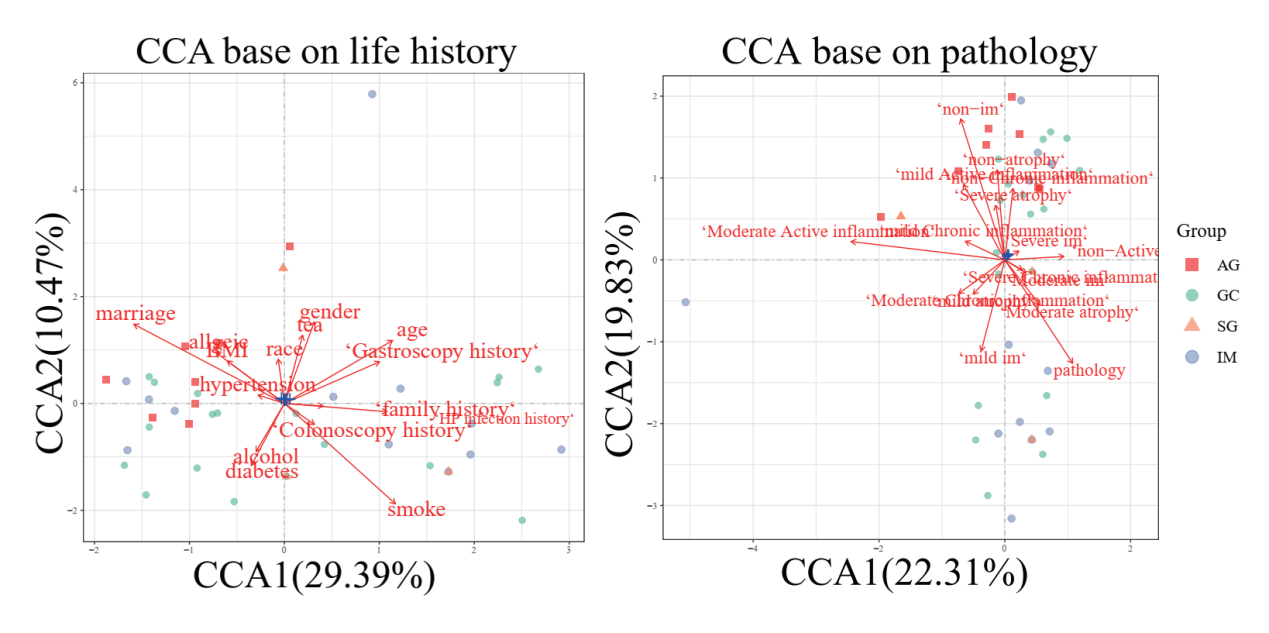


**Figure S4.** CCA plots showing the associations between bulk RNA-seq transcriptome features and life history (left) and pathology features (right).


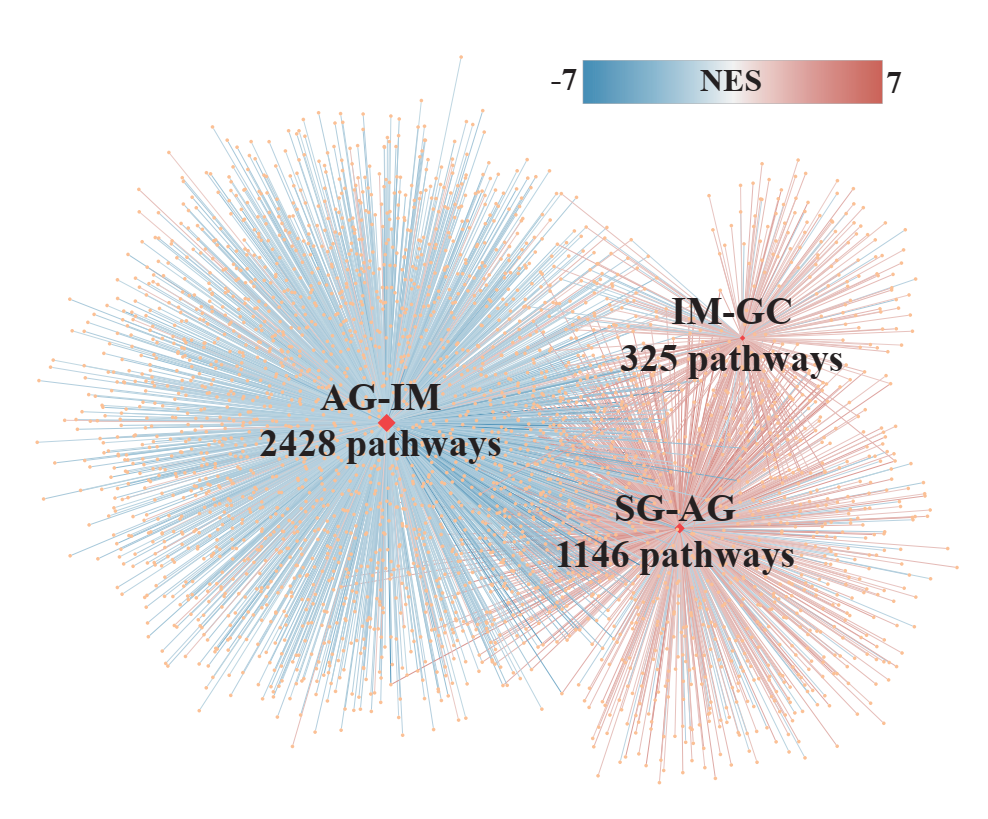


**Figure S5.** Network illustrated the number of significantly enriched gene sets by Gene set enrichment analysis based on the histone modification genes and their co-expressing genes in bulk RNA-seq transcriptome. The color and the width represented the scores of NES calculated by GSEA.


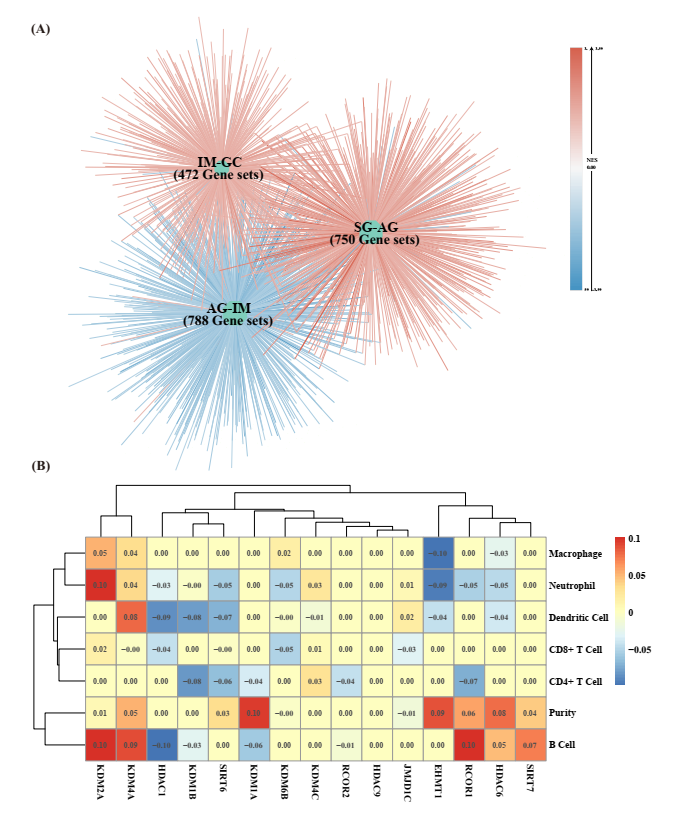


**Figure S6.** The associations between HMGs and immune response. (A) Network illustrated the number of significantly enriched gene sets by Gene set enrichment analysis against ImmuneSigDB based on all detected genes in bulk RNA-seq transcriptome. The color and the width represented the scores of NES calculated by GSEA. (B) Heatmap illustrating correlations between HMGs’ expression and abundance of immune infiltrates using TIMER.


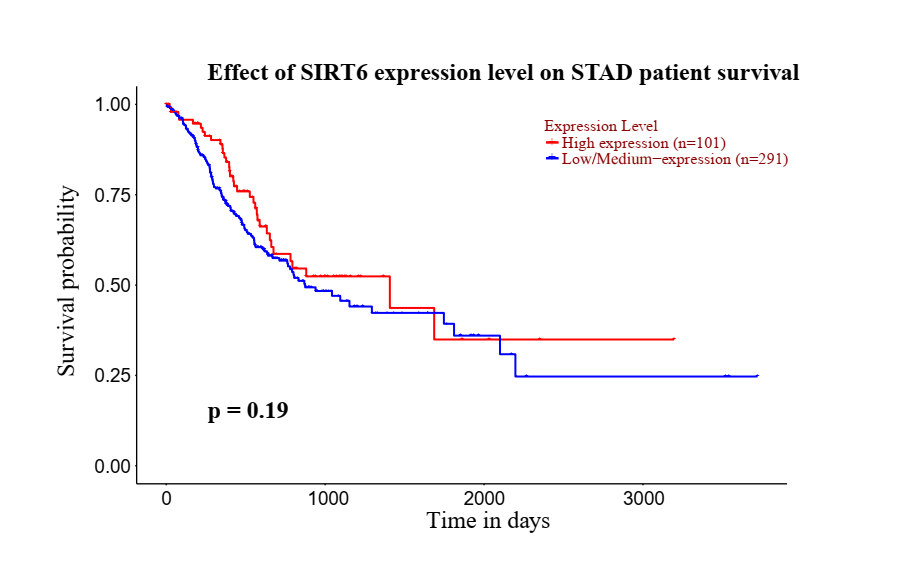


**Figure S7**. Survival analysis predicting the relationship between SIRT6 expression and patient survival.


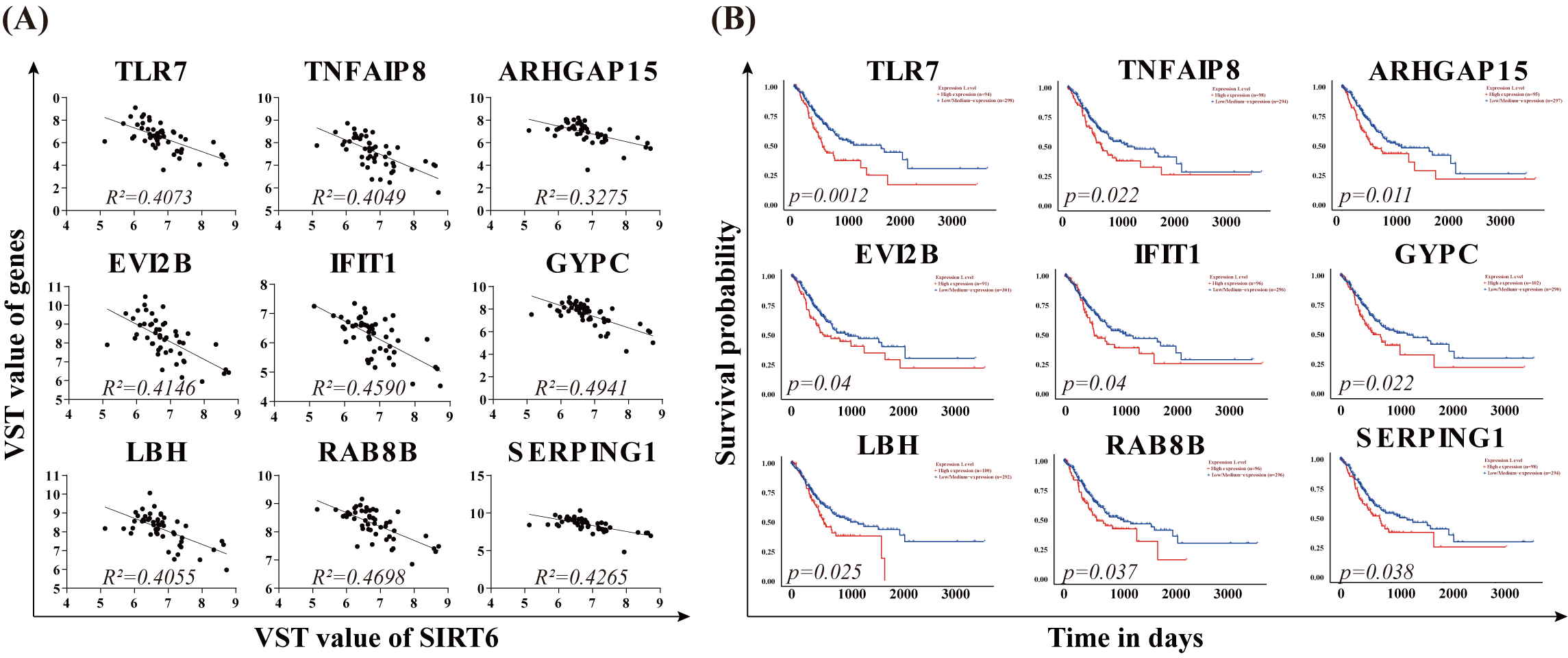


**Figure S8.** (A) Dot plots showing top 9 genes negatively correlated with SIRT6 across all samples. (B) The survival analysis of top 9 genes negatively correlated with SIRT6.


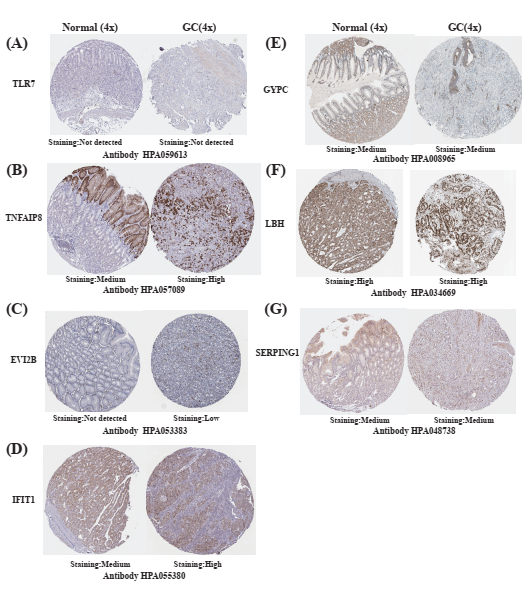


**Figure S9.** The exploration of genes’ immunostaining in control and GC tissues in the Human Protein Atlas. The genes were those negatively correlated with SIRT6 in Figure 7.

Reference

Allis, C. D., S. L. Berger, J. Cote, S. Dent, T. Jenuwien, T. Kouzarides, L. Pillus, D. Reinberg, Y. Shi, R. Shiekhattar, A. Shilatifard, J. Workman and Y. Zhang (2007). "New nomenclature for chromatin-modifying enzymes." Cell **131**(4): 633-636.

Carafa, V., D. Rotili, M. Forgione, F. Cuomo, E. Serretiello, G. S. Hailu, E. Jarho, M. Lahtela-Kakkonen, A. Mai and L. Altucci (2016). "Sirtuin functions and modulation: from chemistry to the clinic." Clin Epigenetics **8**: 61.

Ciccone, D. N., H. Su, S. Hevi, F. Gay, H. Lei, J. Bajko, G. Xu, E. Li and T. Chen (2009). "KDM1B is a histone H3K4 demethylase required to establish maternal genomic imprints." Nature **461**(7262): 415-418.

Fournier, M., M. Orpinell, C. Grauffel, E. Scheer, J. M. Garnier, T. Ye, V. Chavant, M. Joint, F. Esashi, A. Dejaegere, P. Gonczy and L. Tora (2016). "KAT2A/KAT2B-targeted acetylome reveals a role for PLK4 acetylation in preventing centrosome amplification." Nat Commun **7**: 13227.

Huang, C., Y. Xiang, Y. Wang, X. Li, L. Xu, Z. Zhu, T. Zhang, Q. Zhu, K. Zhang, N. Jing and C. D. Chen (2010). "Dual-specificity histone demethylase KIAA1718 (KDM7A) regulates neural differentiation through FGF4." Cell Res **20**(2): 154-165.

Katoh, M. and M. Katoh (2007). "Comparative integromics on JMJD1C gene encoding histone demethylase: conserved POU5F1 binding site elucidating mechanism of JMJD1C expression in undifferentiated ES cells and diffuse-type gastric cancer." Int J Oncol **31**(1): 219-223.

Kramer, J. M., K. Kochinke, M. A. Oortveld, H. Marks, D. Kramer, E. K. de Jong, Z. Asztalos, J. T. Westwood, H. G. Stunnenberg, M. B. Sokolowski, K. Keleman, H. Zhou, H. van Bokhoven and A. Schenck (2011). "Epigenetic regulation of learning and memory by Drosophila EHMT/G9a." PLoS Biol **9**(1): e1000569.

Kuang, J., L. Chen, Q. Tang, J. Zhang, Y. Li and J. He (2018). "The Role of Sirt6 in Obesity and Diabetes." Front Physiol **9**: 135.

Li, B., Y. Zheng and L. Yang (2019). "The Oncogenic Potential of SUV39H2: A Comprehensive and Perspective View." J Cancer **10**(3): 721-729.

Pandzic, T., V. Rendo, J. Lim, C. Larsson, J. Larsson, I. Stoimenov, S. Kundu, M. A. Ali, M. Hellström, L. He, A. M. Lindroth and T. Sjöblom (2017). "Somatic PRDM2 c.4467delA mutations in colorectal cancers control histone methylation and tumor growth." Oncotarget **8**(58): 98646-98659.

Piao, L., M. Nakakido, T. Suzuki, N. Dohmae, Y. Nakamura and R. Hamamoto "Automethylation of SUV39H2, an oncogenic histone lysine methyltransferase, regulates its binding affinity to substrate proteins." (1949-2553 (Electronic)).

Piao, L., X. Yuan, M. Zhuang, X. Qiu, X. Xu, R. Kong and Z. Liu (2019). "Histone methyltransferase SUV39H2 serves oncogenic roles in osteosarcoma." Oncol Rep **41**(1): 325-332.

Rivera, C., H. G. Lee, A. Lappala, D. Wang, V. Noches, M. Olivares-Costa, M. Sjoberg-Herrera, J. T. Lee and M. E. Andres (2022). "Unveiling RCOR1 as a rheostat at transcriptionally permissive chromatin." Nat Commun **13**(1): 1550.

Schizas, D., A. Mastoraki, L. Naar, D. I. Tsilimigras, I. Katsaros, V. Fragkiadaki, G.-S. Karachaliou, N. Arkadopoulos, T. Liakakos and D. Moris (2020). "Histone Deacetylases (HDACs) in Gastric Cancer: An Update of their Emerging Prognostic and Therapeutic Role." Current Medicinal Chemistry **27**(36): 6099-6111.

Shen, X., P. Li, Y. Xu, X. Chen, H. Sun, Y. Zhao, M. Liu and W. Zhang (2017). "Association of sirtuins with clinicopathological parameters and overall survival in gastric cancer." Oncotarget **8**(43): 74359-74370.

Thalhammer, A., J. Mecinovic, C. Loenarz, A. Tumber, N. R. Rose, T. D. Heightman and C. J. Schofield (2011). "Inhibition of the histone demethylase JMJD2E by 3-substituted pyridine 2,4-dicarboxylates." Org Biomol Chem **9**(1): 127-135.

Upadhyay, G., A. H. Chowdhury, B. Vaidyanathan, D. Kim and S. Saleque (2014). "Antagonistic actions of Rcor proteins regulate LSD1 activity and cellular differentiation." Proc Natl Acad Sci U S A **111**(22): 8071-8076.

Wang, Y., Q. Wu, P. Yang, C. Wang, J. Liu, W. Ding, W. Liu, Y. Bai, Y. Yang, H. Wang, S. Gao and X. Wang (2016). "LSD1 co-repressor Rcor2 orchestrates neurogenesis in the developing mouse brain." Nat Commun **7**: 10481.

Wilkins, S. E., M. S. Islam, J. M. Gannon, S. Markolovic, R. J. Hopkinson, W. Ge, C. J. Schofield and R. Chowdhury (2018). "JMJD5 is a human arginyl C-3 hydroxylase." Nat Commun **9**(1): 1180.

Witt, O., H. E. Deubzer, T. Milde and I. Oehme (2009). "HDAC family: What are the cancer relevant targets?" Cancer Lett **277**(1): 8-21.

Yang, P., Y. Wang, J. Chen, H. Li, L. Kang, Y. Zhang, S. Chen, B. Zhu and S. Gao (2011). "RCOR2 is a subunit of the LSD1 complex that regulates ESC property and substitutes for SOX2 in reprogramming somatic cells to pluripotency." Stem Cells **29**(5): 791-801.
